# Allergen-Induced C5a/C5aR1 Axis Activation in Pulmonary CD11b^+^ cDCs Promotes Pulmonary Tolerance through Downregulation of CD40

**DOI:** 10.3390/cells9020300

**Published:** 2020-01-26

**Authors:** Konstantina Antoniou, Fanny Ender, Tillman Vollbrandt, Yves Laumonnier, Franziska Rathmann, Chandrashekhar Pasare, Harinder Singh, Jörg Köhl

**Affiliations:** 1 Institute for Systemic Inflammation Research, University of Lübeck, 23562 Lübeck, Germany; konstantinasantoniou@gmail.com (K.A.); fanny.ender@uksh.de (F.E.); yves.laumonnier@uksh.de (Y.L.); franziska.rathmann@uksh.de (F.R.); 2 Cell Analysis Core, University of Lübeck, 23562 Lübeck, Germany; tillman.vollbrandt@uksh.de; 3 Airway Research Center North, Member of the German Center for Lung Research (DZL), 23562 Lübeck, Germany; 4 Division of Immunobiology, Cincinnati Children’s Hospital Medical Center and University of Cincinnati College of Medicine, Cincinnati, OH 45229, USAharinder@pitt.edu (H.S.)

**Keywords:** allergic asthma, complement, conventional dendritic cell, C5a receptor, CD40, house-dust-mite

## Abstract

Activation of the C5/C5a/C5a receptor 1 (C5aR1) axis during allergen sensitization protects from maladaptive T cell activation. To explore the underlying regulatory mechanisms, we analyzed the impact of C5aR1 activation on pulmonary CD11b^+^ conventional dendritic cells (cDCs) in the context of house-dust-mite (HDM) exposure. BALB/c mice were intratracheally immunized with an HDM/ovalbumin (OVA) mixture. After 24 h, we detected two CD11b^+^ cDC populations that could be distinguished on the basis of C5aR1 expression. C5aR1^−^ but not C5aR1^+^ cDCs strongly induced T cell proliferation of OVA-reactive transgenic CD4^+^ T cells after re-exposure to antigen in vitro. C5aR1^−^ cDCs expressed higher levels of MHC-II and CD40 than their C5aR1^+^ counterparts, which correlated directly with a higher frequency of interactions with cognate CD4^+^ T cells. Priming of OVA-specific T cells by C5aR1^+^ cDCs could be markedly increased by in vitro blockade of C5aR1 and this was associated with increased CD40 expression. Simultaneous blockade of C5aR1 and CD40L on C5aR1^+^ cDCs decreased T cell proliferation. Finally, pulsing with OVA-induced C5 production and its cleavage into C5a by both populations of CD11b^+^ cDCs. Thus, we propose a model in which allergen-induced autocrine C5a generation and subsequent C5aR1 activation in pulmonary CD11b^+^ cDCs promotes tolerance towards aeroallergens through downregulation of CD40.

## 1. Introduction

Allergic asthma is an inflammatory disease of the airways that develops in response to harmless aeroallergens such as the ones from house dust mite (HDM). Dendritic cells (DCs) have been shown to play a key role for the development of maladaptive Th2/Th17 responses in allergic asthma [1]. DCs were initially described as a homogeneous population of CD11c^+^ MHC-II^+^ cells [2]. Ontogenetic studies provided detailed insights into the development of distinct DC lineages. Based on such studies, at least four DC subtypes with distinct functions can be differentiated [3]. In the lung, these include CD11b^+^ and CD103^+^ cDCs, plasmacytoid (p)DCs and, under inflammatory conditions, monocyte-derived cDCs (mo-DCs) [3,4]. Among those, CD11b^+^ CD64^−^ cDCs are critical for the induction of maladaptive Th2/Th17 immune responses [4,5]. Mechanistically, they take up allergens and migrate to the draining lymph nodes, shortly after allergen exposure through activation of C-chemokine receptor-7 (CCR7). CCR7 is upregulated on activated DCs and is involved in the migration of DCs to the lymph nodes [6].

In addition to cDC stimulation, aeroallergens such as HDM drive the activation of the complement system during the early phase of the disease. Complement is already activated under steady state conditions, but this activation is significantly increased upon allergen exposure [7,8]. During allergen-mediated complement activation, the anaphylatoxin C5a is generated and exerts its function mainly through the G-protein coupled receptor, C5aR1. C5a can also be directly generated by HDM-associated proteases which cleave C5 into C5a and C5b in vitro in a dose and time-dependent manner [9]. Additionally, alveolar macrophages can cleave C5 into C5a using a serine protease [10]. C5a has distinct roles in the pathogenesis and the pathology of asthma, depending on the conditions under which it is generated, the cell-types that become activated and the stage of the disease [11,12,13,14,15,16].

Several reports provide evidence that C5a has a regulatory role in the development as well as the magnitude of adaptive immune responses. More precisely, the available data suggest that the C5a/C5aR1 signaling axis controls allergic asthma at the DC/T cell interface. Thus far, the majority of the data suggest that activation of C5aR1 by the C5/C5a axis protects from the development of a Th2 immune response during sensitization phase. In more detail, C5^−^/^−^ mice suffer from increased airway hyperresponsiveness (AHR) and pulmonary inflammation [17,18,19]. In vivo blockade of C5aR1 during the sensitization phase in OVA- and HDM-induced experimental allergic asthma models resulted in increased levels of Th2 cytokines and an overall increased airway inflammation [11]. The mechanisms underlying the protective effect of C5aR1 during allergen sensitization are still ill-defined. C5aR1 targeting has been reported to control the balance between CD11b^+^ cDCs and pDCs during allergen sensitization [11]. However, it is still unclear whether C5aR1 signaling has a direct impact on the activation of pulmonary CD11b^+^ cDCs and consequent prime of allergen-specific T cells.

Here, we assessed C5aR1 expression on primary pulmonary CD11b^+^ cDCs from either naïve BALB/c mice or mice treated once with a mixture of HDM and OVA. We found two distinct CD11b^+^ cDC populations that were either C5aR1^+^ or C5aR1^−^. We determined the potency of C5aR1^+^ and C5aR1^−^ CD11b^+^ cDCs to drive T cell proliferation and differentiation ex vivo in cells purified by Fluorescence-activated cells sorting (FACS). Although C5 production and C5a generation by both cDC populations was similar, we found that C5aR1^+^ cDCs had a markedly lower potency to stimulate T cell proliferation as compared with C5aR1^−^ cDCs. Mechanistically, we discovered that C5aR1 signaling negatively regulated expression of CD40 on MHC-II^lo^ CD11b^+^ cDCs. C5aR1^−^ cDCs that were unable to sense C5a had higher expression of MHC-II and CD40 suggesting that C5a dampens allergen specific T cell response by controlling CD40 expression on CD11b^+^MHC-II^lo^ pulmonary DCs.

## 2. Materials and Methods

### 2.1. Mice

Wildtype (WT) female BALB/c mice were purchased from Charles River laboratories and used at 8–12 weeks of age. The DO11.10RAG2^−/−^ mice on the BALB/c background were bred and kept in the specific-pathogen free (SPF) facility of the University of Lübeck according to institutional and national guidelines. The studies were reviewed and approved by the Animal Care and Use Committee from the Schleswig Holstein state authorities—Ministerium für Landwirtschaft, Energiewende und ländliche Räume, Kiel, Germany (39 (75-6/16), 39 (44-5/18) and 39_2017-0301).

### 2.2. Model of Combined HDM/OVA-Mediated Allergen Exposure

WT BALB/c mice were anesthetized and a mixture of HDM/OVA (100 μg/40 μg) allergens dissolved in PBS was intra-tracheally (i.t.) administered to the throat in a total volume of 50 μL. Twenty-four hours after HDM/OVA exposure, the mice were euthanized, and the lungs were harvested for further analysis as described [20,21].

### 2.3. Pulmonary Cell Isolation

Liberase TL (Roche) 0.25 mg/mL and DNase I 0.5 mg/mL (Sigma-Aldrich, Munich, Germany) digests of the lungs were prepared to obtain single lung cell suspensions as described previously [20,21]. Briefly, the lung tissue-containing cell strainer was transferred to a 50 mL Falcon tube. The single cell suspension was prepared by mechanical disruption of the lungs using a 5 mL syringe stamp and additional 10 mL complete medium (complete medium: RPMI 1640 supplemented with 10% Fetal bovine serum (FBS, PAA Laboratories, Pasching, Austria) heat inactivated, 100 Units/mL Penicillin, 100 µg/mL Streptomycin, 2 mM l-Glutamine) in the presence of 0.5 mg/mL DNase. The smoothing step was repeated twice with a subsequent step of washing with 5 mL of complete medium. The cell suspension was centrifuged for 10 min at 350× *g* at 4 °C. The supernatant was discarded, and the pellet was re-suspended in 3 mL red blood cell lysis buffer for 3 min at room temperature (RT). The lysis was stopped by adding 30 mL of PBS and the cell suspension was centrifuged for 5 min at 400× *g* at 4 °C. After cell counting in a Neubauer counting chamber, the single cell suspension was used for further analysis.

### 2.4. Flow Cytometric Analysis and Antibodies

Phenotypic characterization of cells was performed on a BD LSRII flow cytometer or an ARIA III cell sorter using published gating strategies [4,22]. Monoclonal phycoerythrin (PE) CF^TM^594-labeled Ab against CD11C (clone HL3), Brilliant Violet (BV) 421 or PE Ab against SiglecF (E50-2440), V450- labeled Ab against Ly6G (1A8) and unlabeled anti-C5a (clone I52-1486) were purchased from BD Biosciences. Fluorescein-5-isothiocyanate (FITC)- or allophycocyanin (APC)-Cy7-labeled Ab against MHC-II (clone M5/114.15.2), monoclonal PE-labeled Ab against CD64 (clone X54-5/7.1), Peridinin-chlorophyll proteins (PerCP), Cy5.5 against CD103 (clone 2E7), PE Cy7 against CD88 (clone 20/70), PE against CD24 (clone M1/69), PE against CD301 (clone LOM-14), APC against CD86 (clone GL-1), APC against OX40L (clone RM134L) were purchased from Biolegend (London, United Kingdom). EFluor (eF)450-labeled Abs against CD19 (1D3), CD3e (145-2C11) or CD49b (DX5); APC-labeled Ab against CD11c (N418), APC-labeled Ab against CD40 (IC10), APC-labeled Ab against Foxp3 (FJK-16s), PE-labeled Ab against IL-13 (eBio13A), APC-e780 labeled Ab against MHC-II (M5/114.15.2), PE-Cy7 labeled Ab against CD4 (RM4-5), APC-labeled Ab against CD11b (clone M1/70), APC-labeled Ab against CD80 (clone 16-10A1), APC-labeled Ab against interferon (IFN)γ (XMG1.2) and APC-e780- and eF450-labeled fixable viability dye were obtained from eBioscience (Vienna, Austria). APC-labeled Ab against IL17A (TC11-18H10) was purchased from Miltenyi Biotec (Bergisch-Gladbach, Germany). The C5-specific Ab (BB5.1) was purchased from Hycult Biotech (Uden, The Netherlands) and labeled with AF647 using kit A20186 from Thermo Fisher Scientific (Dreieich, Germany). The blocking antibodies InVivoMAb anti-mouse CD40L (CD154) (MR-1) and InVivoMAb anti-mouse MHC-II (I-A/I-E) (M5/114) were from BioXCell (West Lebanon, NH, USA). The anti-mouse C5aR1 (CD88)-blocking mAb 20/70 was purchased from Hycult Biotech (Uden, The Netherlands).

### 2.5. Isolation and CFSE-Labelling of OVA-Specific T Cell Receptor Transgenic CD4^+^ T Cells

OVA-specific T cell receptor (TCR) transgenic (tg) CD4^+^ T cells were isolated from the spleen of DO11.10 RAG2^−/−^ mice. Mice were sacrificed by CO_2_ and cervical dislocation. The spleen was removed and placed in ice-cold PBS. The single cell suspension was prepared by mechanical disruption of the spleen using a 5 mL syringe stamp and additional 10 mL of PBS in the presence of 0.5 mg/mL DNase. The spleen cells were then centrifuged for 10min at 350× *g* at RT. The negative isolation of CD4^+^ T cells was done with the CD4^+^ T cell isolation kit from Miltenyi Biotec (Bergisch-Gladbach, Germany) according to the manufacturer’s instruction. Briefly, the cell pellet was resuspended in 400 μL of MACS buffer (MACS BSA stock solution (Miltenyi Biotec, Bergisch-Gladbach, German) diluted 1:20 in PBS) and then incubated for 5 min at 4 °C in 100 μL of biotin-antibody cocktail. The biotin antibody cocktail comprised biotin-conjugated antibodies against CD8a, CD11b, CD11c, CD19, CD45R (B220), CD49b (DX5), CD105, Anti-MHC-class II, Ter 119 and TCRγ/δ as primary labelling reagent. The cell suspension was then washed with 200 μL of MACS buffer and incubated for 10 min at 4 °C in 200 μL anti-biotin microbeads. The cells were washed with a 10 ml MACS buffer and centrifuged for 5 min at 400× *g* at RT. The pellet was re-suspended in MACS buffer followed by magnetic cell separation using the MACS cell separation device. The cells were counted and some of them were stained with an anti-CD4 Ab coupled to PE-Cy7 to check for purity, which was always >90%. For 6-Carboxyfluorescein (CFSE) labeling of the CD4^+^ T cells, the CellTrace CFSE cell proliferation kit (Thermo Fischer Scientific, Schwerte, Germany) was used according the manufacturer’s instructions. Briefly, after magnetic separation, the cells were centrifuged for 5 min at 400× *g* at RT and the cell pellet was re-suspended in 500 μL of the labeling buffer (5 mL of pre-warmed PBS + 0.5 μL of 10 mM CFSE). The cells were incubated in the labeling buffer for 10 min at 37 °C in the dark. The reaction was stopped by adding 1 mL ice-cold PBS and incubating the cells for 1min on ice. The cells were then centrifuged, and the pellet re-suspended in complete medium. The labeling success was determined by flow cytometry. After successful CFSE labeling, CD4^+^ T cells were added to a 96-well plate to which CD11b^+^ cDCs were plated the day before.

### 2.6. Co-Culture of Sorted cDCs with Naïve CD4^+^ T Cells from DO11.10 RAG2^−/−^ Mice

C5aR1^−^ and C5aR1^+^ CD11b^+^ cDCs were obtained 24 h after a one step HDM/OVA or HDM exposure. Living SiglecF^−^ lin^−^ CD11c^+^ MHC-II^+^ CD103^−^ CD11b^+^ CD64^−^ C5aR1^+^ cDCs and SiglecF^−^ lin^−^ CD11c^+^ MHC-II^+^ CD103^−^ CD11b^+^ CD64^−^ C5aR1^−^ cDCs were sorted using a BD FACSAria III sorter, and co-cultured with CFSE-labeled OVA-tg CD4^+^ T cells, isolated by magnetic selection (Miltenyi Biotec, Bergisch-Gladbach, Germany) from DO11.10RAG2^−/−^ mice at a ratio of 1:2.5, as previously described [14,23]. Briefly, the cDC subsets were seeded in a 96-well U-bottom plate, cultured in complete medium supplemented with 20 ng/mL granulocyte-macrophage-colony stimulating factor (GM-CSF) and pulsed overnight with 10 μM OVA (grade III). CFSE-labelled OVA-tg CD4^+^ T cells were added the next day at a ratio of 1:2.5 (cDC:T cell). After 4 days, the cells were transferred to a 1.5 mL Eppendorf tube and centrifuged for 10 min at 350× *g* at 4 °C. The supernatant was used for further analysis. In some experiments, C5aR1 in C5aR1^+^ cDCs was blocked by C5aR1-specific mAb 20/70 (5 μg/mL). Furthermore, in some experiments, C5aR1^+^ cDCs were additionally treated with CD40L-specific mAb MR-1 (5 μg/mL). In another set of experiments, MHC-II was targeted with different concentrations (100 pg/mL or 10 pg/mL) of MHC-II-specific mAb M5/114. In all setups, appropriate isotype control antibodies were used for comparison. Additionally, in some experiments CD11b^+^ C5aR1^+^ cDCs were pulsed with OVA^323−339^ peptide at different concentrations (5 μg/mL, 500 ng/mL, 50 ng/mL, 20 ng/mL or 5 ng/mL) instead of whole OVA antigen in the presence or absence of C5aR1-specific mAb 20/70. Finally, we used a setup in which CD11b^+^C5aR1^−^ cDCs were pulsed with OVA^323−339^ peptide at different concentrations instead of whole OVA antigen in the presence or absence of CD40L-specific mAb MR-1.

### 2.7. Determination of Cytokine Production and Foxp3 Expression in OVA-Specific TCR tg CD4^+^ Cells

Intracellular T cell-staining was performed as described [23]. Briefly, after 4 days of co-culture with C5aR1^+^ or C5aR1^−^ cDCs, CD4^+^ OVA-specific TCR tg T cells were stimulated with 500 ng/mL ionomycin and 50 ng/mL phorbol 12-myristate 13-acetate (PMA). The Golgi-apparatus was simultaneously blocked with Brefeldin A and Monensin for 4 h at 37 °C, 5% CO_2_. Afterwards, the cells were harvested and stained with a fixable viability dye, CD4, IL-13, IL-17A, IFN-γ or FOXP3 using a Foxp3 staining buffer set according to the manufacturer’s instructions (eBioscience, Vienna Austria).

### 2.8. Saponin-Based Permeabilization Approach

For intracellular staining of the complement fragments C5 and C5a, C5aR1- and C5aR1^+^ CD11b^+^ cDCs as well as CD4^+^ T cells were first stained with LIVE/DEAD fixable dead cell stain (Invitrogen/Thermo Fischer Scientific, Schwerte, Germany). Living cells were then fixed in 1.5% paraformaldehyde and resuspended in a saponin (0.2%) buffer containing 20% FCS. After 30 min, cells were stained either with the AF647-labeled BB5.1 C5 or with the I52-1486 anti-C5a Ab in saponin (0.2%) buffer containing 20% FCS. In case of the C5a staining, after washing, cells were stained with a goat anti-rat IgG1 coupled to PE. The cells were washed twice and analyzed on the LSRII flow cytometer.

### 2.9. Laser Scanning Microscopy to Assess Dendritic Cell T Cell Interactions

To determine the interaction between CD11b^+^ cDCs and T cells, the FACS-purified sensitized CD11b^+^ C5aR1^+^cDCs and CD11b^+^ C5aR1^−^ cDCs were seeded in a 96-well U-bottom plate in complete medium supplemented with 20 ng/mL GM-CSF and pulsed with 10 μM OVA. Eighteen hours later, the cells were collected into an Eppendorf tube and labeled with the red fluorescent dye PKH26 (Merck, Darmstadt, Germany) (membrane labeling) according to manufacturer’s instructions. Briefly, the collected cells were washed with 1 mL pre-warmed serum-free medium (pure RPMI 1640) and centrifuged for 5 min at 400× *g*. The supernatant was aspirated. Then, 100 μL of diluent C, which is an aqueous solution designed to maintain cell viability, while maximizing dye solubility and staining efficiency during the labeling step were added to 1 × 10^5^ cells. The diluent was mixed with the cells by gently pipetting up and down. Immediately prior to staining, the dye solution was prepared by adding 0.4 μL of PKH26 ethanolic solution dye to 100 μL Diluent C in a separate Eppendorf tube. Then, the dye was quickly pipetted into the Eppendorf tube, which already contained the cells. Subsequently, the cells were mixed with the dye by gently pipetting the cell solution up and down. Afterwards, the cells were incubated with the dye for 5 min at RT in the dark with periodic mixing. The staining was stopped by adding 1 mL of pre-warmed complete medium. The sample was centrifuged at 400× *g* for 10 min at RT. The supernatant was discarded, the cells were washed in 1 mL pre-warmed complete medium and centrifuged again at 400× *g* for 10 min at RT. The supernatant was aspirated, and the pellet was resuspended in 50 μL of pre-warmed complete medium. The OVA tg CD4^+^ T cells were negatively isolated by MACS and labeled with CFSE according to the manufacturer’s instructions. CFSE-labeled-CD4^+^ T cells (1 × 10^5^) were resuspended in 50 μL of pre-warmed complete medium and transferred together with the PKH26-labeled cDCs into an Eppendorf tube. One end of the incubation chamber was unscrewed, and the mixture of DC-T cells was added by continuously pipetting to avoid bubbles. Afterwards, 700 μL of pre-warmed complete medium was slowly added to both ends of the chamber to avoid perturbation of the cells. The cells were incubated for 30 min at 37 °C and subsequently transferred to the Olympus FV1000 IX81 confocal microscope to assess the DC-T cell interactions. The DC-T cell interactions were recorded for 5 h by taking one picture per minute.

### 2.10. Statistical Analysis

Statistical analysis was performed using GraphPad Prism version 7 (GraphPad Software, Inc., LaJolla, CA, USA). Normal distribution of data was tested using the Kolmogorov-Smirnov and D’Agostino-Pearson tests. When groups were normally distributed, statistical differences between two groups were analyzed by unpaired t test. If more than two groups were evaluated, the groups were first analyzed by an analysis of variance (one-way ANOVA), and in case of significance, followed by a Tukey’s test. A *p*-value < 0.05 was considered as statistically significant, * represents *p* < 0.05; ** represents *p* < 0.01; *** represents *p* < 0.001 and **** represents *p* < 0.0001.

## 3. Results

### 3.1. Pulmonary CD11b^+^cDCs Are a Heterogeneous DC Population

First, we aimed at characterizing pulmonary CD11b^+^ cDCs in WT mice. In the first step, we excluded macrophages and eosinophils as SiglecF^+^ cells. Within the SiglecF^−^ fraction, we excluded B cells (CD19), T cells (CD3e), NK cells (CD49b) and neutrophils (Ly6G) as lineage^−^ cells. Among the lineage^−^ cells, DCs were identified as CD11C^+^ MHC-II^hi^ cells. Using CD103 and CD11b as markers, we further subdivided DCs into CD103^+^ CD11b^−^ cDCs and CD103^−^ CD11b^+^ cells comprising CD11b^+^ CD64^−^ cDCs and CD11b^+^ CD64^+^ mo-DCs.

**Figure 1:**
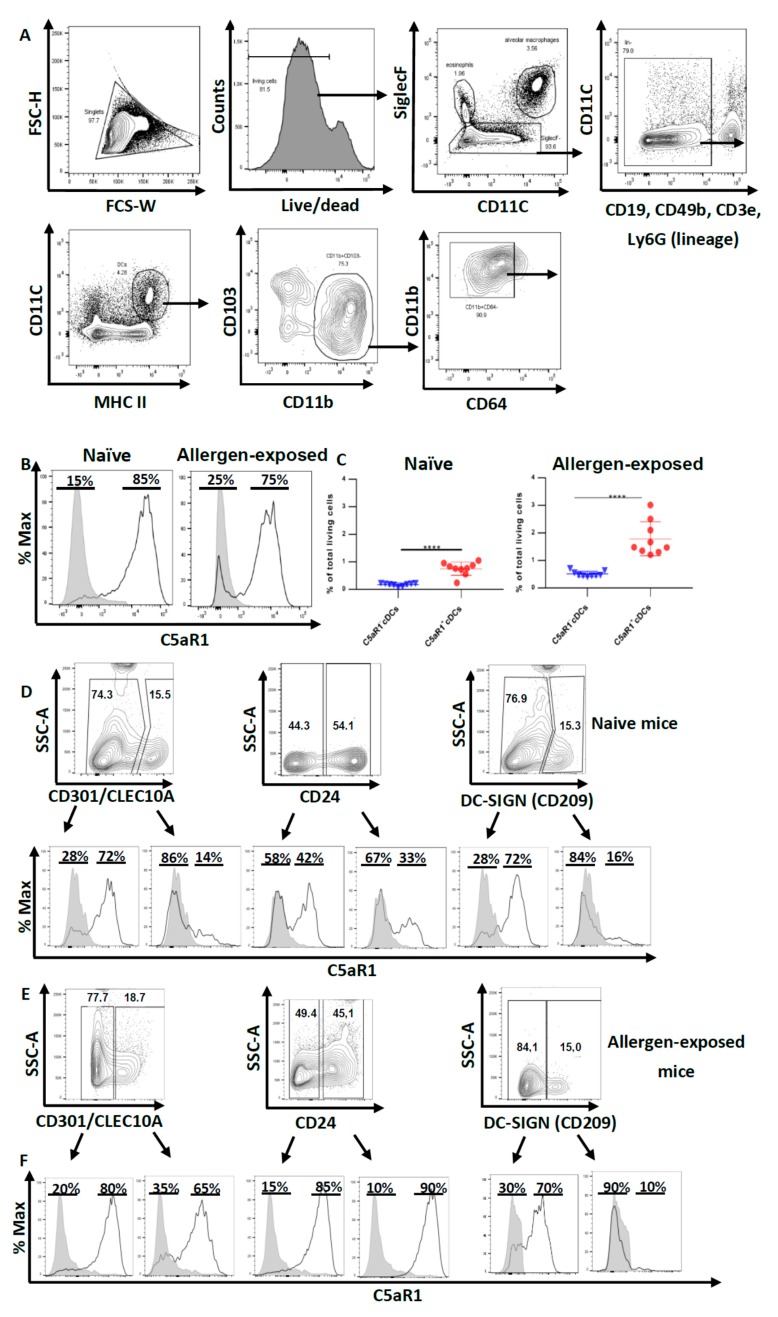
Pulmonary CD11b^+^ cDCs are a heterogeneous cDC population. (**A**) Gating strategy to identify pulmonary CD11b^+^ cDCs in WT naïve and allergen-exposed BALB/c mice. CD11b^+^ cDCs were identified as SiglecF^−^ lin^−^ CD11C^+^ MHC-II^hi^ CD103^−^ CD11b^+^ CD64^−^ cells. (**B**) Histograms showing the expression of C5aR1 in pulmonary CD11b^+^ cDCs from both naive mice (left panel) and animals exposed 1× i.t. to HDM/OVA (right panel). The data shown in the histograms are representative of 10 mice. (**C**) Frequencies of pulmonary CD11b^+^ C5aR1^−^ and CD11b^+^ C5aR1^+^ cDCs in naïve (left panel) and mice immunized 1× i.t. with HDM/OVA (right panel). Shown are the frequencies of the two subsets within total lung cells as the mean ± standard error of the mean (SEM), *n* = 10. Differences between groups were determined by unpaired t-test; **** *p* < 0.0001. (**D**,**E**) Dot plots showing the expression of CD301, CD24 and CD209 in pulmonary CD11b^+^ cDCs from (**D**) naive or (**E**) mice exposed 1× i.t. to HDM/OVA. The histograms show the frequency of C5aR1^+^ cells within the CD11b^+^ cDC population that were either CD301, CD24 or CD209 positive or negative. (**F**) Quantitative evaluation of the frequency of C5aR1^+^ or C5aR1^−^ cells within the subsets of CD301, CD24 or CD209 positive or negative cells. The gray histograms represent Fluorescence minus one (FMO) controls.

Based on this gating strategy, we sorted CD11b^+^ cDCs by FACS (Figure 1A). Next, we determined the expression of C5aR1 in such cells as previous data from C57BL/6 GFP-C5aR1 knock-in mice suggested that this pulmonary cDC subpopulation is heterogeneous regarding the expression of C5aR1 [22]. We found that 85% of the CD11b^+^ cDCs cells were C5aR1^+^ and 15% were C5aR1^-^ under steady-state conditions (Figure 1B). Interestingly, we noticed that 24 h after i.t. HDM/OVA administration, the majority of the CD11b^+^ cDCs was still positive for C5aR1 (75%), but the frequency of C5aR1^−^ cDCs increased to 25% (Figure 1B,C), which corresponded to a frequency of 0.8% for C5aR1^+^ cDCs and 0.1% for C5aR1^−^ cDCs of total lung cells. The 24 h exposure to HDM/OVA resulted in a two-fold increase in the frequency of the C5aR1^+^ subset to 1.6%; the C5aR1^−^ cDCs increased six-fold finally reaching almost 0.6% of all lung cells (Figure 1C).

Given that CD11b^+^ cDCs differ in the expression of C5aR1, we determined the expression of three markers that are expressed on APCs, i.e., the two lectin receptors CD209 (DC-SIGN) and CD301(CLEC10A) and the GPI-linked sialoglycoprotein CD24, which exert pleiotropic functions in different DC subsets [24,25,26]. The expression of these molecules was examined under steady state conditions and after HDM/OVA challenge (Figure 1D–F). Several reports have highlighted that pulmonary DC populations are heterogeneous [27,28,29]. We found that the majority of pulmonary CD11b^+^ cDCs from naïve mice stained negative for CD301 or CD209 (about 75%). In contrast, about 50% of CD11b^+^ cDCs expressed CD24 (Figure 1D). Interestingly, the distribution pattern did not change after one-time HDM/OVA immunization (Figure 1E). Previously, FLT3- and IRF-4-dependent CD11b^+^ CD24^+^ CD64^−^ bone fide cDCs have been differentiated from CSF-1R-dependent CD11b^+^ CD24^−^ CD64^+^ macrophages [30]. In support of this view, the DC-lineage-specific transcription factor Zbtb46 was found to be highly expressed in CD11c^+^ CD11b^+^ CD24^+^ MHC-II^+^ pulmonary DCs [27]. Interestingly, we found that only 50% of the CD11b^+^ CD64^−^ cDCs stained positive for the CD24 marker and about 33% of the CD11b^+^ CD24^+^ CD64^−^ cDCs expressed C5aR1 whereas two thirds of the CD11b^+^ CD24^+^ CD64^−^ cDCs did not. In response to allergen exposure, the majority of CD11b^+^ CD24^+^ (90%) or CD11b^+^ CD24^−^ (85%) cDCs expressed C5aR1, suggesting allergen-driven upregulation of C5aR1 in particular in the CD24^+^ fraction of cDCs (Figure 1E,F). Similarly, we noticed a strong upregulation of C5aR1 in the CD11b^+^ CD301^+^ subset of cDCs (from 14% to 65%) (Figure 1E,F). In contrast, C5aR1 expression was minor in the CD11b^+^ DC209^+^ subset of cDCs and was not affected by allergen immunization. Together, these findings support the view that the pulmonary CD11b^+^ CD64^−^ cDCs compartment is heterogeneous and that C5aR1 is expressed to different degrees in the different subsets of pulmonary CD11b^+^ CD64^−^ cDCs that express CD24, CD301 or CD209.

### 3.2. Impact of C5aR1^+^ and C5aR1^−^ cDCs on the Proliferation and Differentiation of OVA-Specific TCR tg CD4^+^ T Cells

In the next set of experiments, we assessed whether the phenotypic differences of CD11b^+^ cDCs were associated with functional differences. First, we evaluated the potency of the C5aR1^+^ and C5aR1^−^ cDC sub-populations to drive OVA-specific CD4^+^ T cell proliferation. More precisely, 24 h after 1 step HDM/OVA immunization, the C5aR1^+^ and C5aR1^−^ cDCs were FACS-purified, in vitro pulsed with OVA and, 18 h later, co-cultured with CFSE-labeled-OVA-specific TCR Tg-CD4^+^ T cells from DO11.10 RAG2^−^/^−^ mice. Four days later, the proliferation was evaluated.

**Figure 2:**
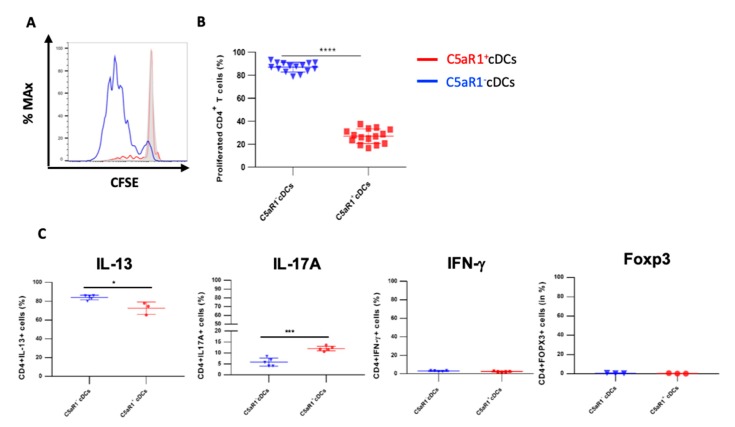
Proliferation and differentiation of OVA-transgenic CD4^+^ T cells in response to co-culture with OVA-pulsed C5aR1^+^ or C5aR1^−^ cDCs. WT BALB/c mice were treated once with a combination of HDM/OVA (100 μg/40 μg) i.t. C5aR1^+^ and C5aR1^−^ CD11b^+^ cDCs from WT mice were FACS-purified from digested lung tissue 24 h after immunization. Both cell populations were co-cultured with CFSE-labeled OVA-specific TCR tg CD4^+^ T cells in the presence of OVA (10 μΜ) and GM-CSF (20 ng/mL) for four days. (**A**) Histogram showing the CFSE signal in OVA-specific TCR Tg CD4^+^ T cells after four days of co-culture with either C5aR1^+^ or C5aR1^−^ cDCs. The grey histogram shows the signal obtained from T cell directly after CFSE labeling. (**B**) Frequency of proliferated OVA-specific TCR tg CD4^+^ T cells, Data shown are the mean ± SEM, *n* = 15 per group. (**C**) On day 4, the differentiation of T cells was evaluated by intra-cellular cytokine staining. Shown is the percentage of proliferated CD4^+^ T cells expressing IL-13, IL-17A, IFN-γ or FOXP3 as the mean ± SEM, *n* = 3–5 per group. Differences between groups were assessed by unpaired t-test; * indicates significant differences between T cell co-cultures of C5aR1^−^ vs. C5aR1^+^ cDCs; * *p* < 0.05, *** *p* < 0.001, **** p < 0.0001.

After four days of co-culture, the percentage of living CD4^+^ T cells was typically between 90–93% in co-cultures with C5aR1^+^ and C5aR1^−^ cDCs, demonstrating that both CD11b^+^ cDC populations promoted T cell survival (data not shown). However, the C5aR1^+^ cDCs had a much lower potency to drive T cell proliferation than their C5aR1^−^ counterparts (Figure 2A,B). While C5aR1^+^ cDCs induced proliferation in 20% of T cells, C5aR1^−^ cDCs stimulated proliferation of almost 90% of T cells. Next, we tested the ability of the two CD11b^+^ cDC populations to induce Th2 and/or Th17 differentiation. C5aR1^−^ and C5aR1^+^ cDCs induced the differentiation of IL-17-producing Th17 cells, although the C5aR1^−^ cDC/T cell co-cultures resulted in a lower frequency of IL-17A^+^ producing T cells (5 ± 3%) when compared to C5aR1^+^ cDC/T cell co-cultures (10 ± 5%; Figure 2C).

Moreover, C5aR1^+^ and C5aR1^−^ cDC/T cell co-cultures resulted in the differentiation of IL-13 producing Th2 cells. The frequency of IL-13-producing Th2 cells was between 70 ± 5% and 80 ± 5% in co-cultures of C5aR1^+^ and C5aR1^−^ with T cells, respectively (Figure 2C). Thus, C5aR1^+^ and C5aR1^−^ cDCs induced a dominant Th2 response, which was slightly more pronounced using C5aR1^−^ cDCs. As expected, both CD11b^+^ cDC subsets hardly induced any IFN-γ-producing Th1 or FOXP3-expressing Treg cells (Figure 2C).

### 3.3. C5aR1^+^ cDCs Express Significantly Lower Levels of MHC-II and CD40 than C5aR1^-^ cDCs after HDM/OVA Exposure

**Figure 3:**
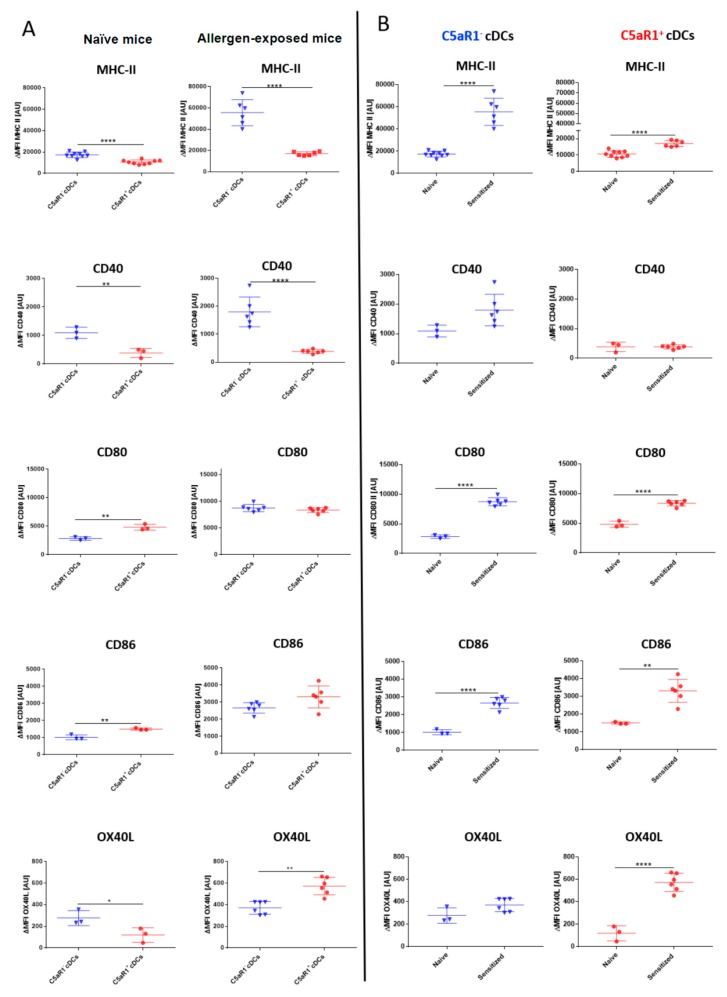
Association of C5aR1 expression on CD11b^+^ cDCs with MHC-II, CD40, CD80, CD86 and OX40L in naïve and HDM/OVA-immunized mice. (**A**) Costimulatory molecule expression in cDCs from either naïve WT mice (left panel) or WT mice treated once i.t. with a mixture of HDM/OVA (100 μg/40 μg) (right panel). Twenty-four hours after the in vivo treatment, the expression of MHC-II and the co-stimulatory molecules CD40, CD80, CD86, and OX40L was evaluated by measuring 10,000 events for each of the two subpopulations. (**B**) Comparison of MHC-II and costimulatory expression levels before and after treatment with HDM/OVA (100 μg/40 μg) in C5aR1^−^ cDCs (left panel) and C5aR1^+^ cDCs (right panel). Shown is the ΔMFI of the expression levels of the examined molecules by the two subsets as mean ± SEM; the ΔMFI is defined as the mean fluorescence intensity of the signal normalized to the FMO control; *n* = 3–6 per group; differences between groups were evaluated by unpaired t-test, * *p* < 0.05; ** *p* < 0.01; **** *p* < 0.0001.

To understand the mechanisms underlying the reduced potency of the C5aR1^+^ cDCs to efficiently induce T cell proliferation, we evaluated the expression of MHC-II and the costimulatory molecules CD40, CD80, CD86 and OX40L in C5aR1^+^ and C5aR1^−^ cDCs under steady-state conditions and 24 h after HDM/OVA exposure (Figure 3). In C5aR1^-^ cDCs from naïve WT mice, MHC-II, CD40 and OX40L expression was significantly higher than that observed in C5aR1^+^ cDCs. In contrast expression of CD80 and CD86 was significantly higher in C5aR1^+^ than in C5aR1^−^ cDCs (Figure 3A, left panel). Similar to the steady state conditions, expression of MHC-II and CD40 was significantly higher in C5aR1^−^ as compared to C5aR1^+^ cDCs 24h after HDM/OVA exposure. CD80 and CD86 expression was similar in both cDC populations, whereas OX40L expression was higher in C5aR1^+^ cDCs than in C5aR1^−^ cDCs after allergen immunization (Figure 3A, right panel). Finally, we compared the expression of each molecule in naïve and allergen-exposed mice. In response to HDM/OVA immunization, MHC-II expression increased in both cell populations (Figure 3B). However, the increase in C5aR1^−^ cDCs was markedly higher than in C5aR1^+^ cDCs. Furthermore, while both CD11b^+^ cDC subsets expressed CD40, the expression of CD40 increased only in the C5aR1^−^ group, whereas it remained stable in C5aR1^+^ cDCs 24 h after HDM/OVA immunization (Figure 3B). CD80 and CD86 expression similarly increased upon HDM/OVA immunization in both cDC subsets (Figure 3B). Finally, we found that HDM/OVA exposure increased the expression of OX40L in C5aR1^+^ but not in C5aR1^−^ cDCs (Figure 3B). Based on these findings, we hypothesized that the impaired potency of C5aR1^+^ cDCs to drive T cell proliferation resulted from the lower MHC-II and CD40 expression as compared with C5aR1^−^ cDCs.

### 3.4. C5aR1^+^ cDCs Interact Less Frequently with OVA-specific TCR tg CD4^+^ T Cells than C5aR1^−^ cDCs

**Figure 4:**
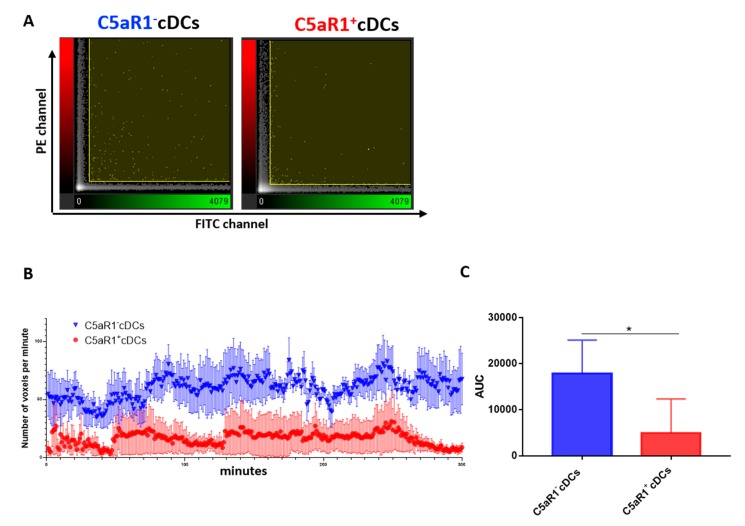
Monitoring of the interaction between OVA-specific TCR tg CD4^+^ T cells and C5aR1^−^ or C5aR1^+^ cDCs. WT mice were treated once with a combination of HDM/OVA (100 μg/40 μg) i.t. C5aR1^+^ and C5aR1^−^ CD11b^+^ cDCs from WT mice were FACS-purified from lung tissue 24 h after allergen exposure. The cDCs were pulsed with the OVA (10 μΜ) and GM-CSF (20 ng/mL) overnight. The following day, they were labeled with PKH26 and transferred in a channel slide together with CFSE-labeled OVA-specific TCR tg CD4^+^ T cells. Using an FV1000 confocal microscope, the interactions between cDCs and T cells were tracked for 300 min (one picture/minute). To visualize the interactions between the two cell populations, the Imaris™ coloc tool was used. It operates simultaneously on two channels (PE and FITC) and measures the degree of overlap between the two channels. (**A**) Intensity histogram of the PE/FITC channels, which reflects the distribution of voxel pair intensities occurring in the two selected channels (a voxel is a unit of graphic information that defines a point in three-dimensional space). The range of intensity pairs considered as colocalized can be defined on the histogram as channel thresholds, marked with the two yellow lines. The voxel numbers in C5aR1^−^ cDCs (left) were higher than in C5aR1^+^ cDCs (right). (**B**) Number of voxels per minute monitored for a time period of 300 min using either C5aR1^−^ cDCs (blue) or C5aR1^+^ cDCs (red), *n* = 4 per group. (**C**) Quantitative evaluation of the curves shown in (**B**). The area under the curve (AUC) was determined for evaluation. Data shown are the mean ± SEM; *n* = 4 per group; differences between groups were evaluated by unpaired t-test, * *p* < 0.05.

The activation of naive T cells by cDCs is mediated by allergen peptide-loaded MHC-II on cDCs that is recognized by the TCR. In addition, interaction of co-stimulatory molecules and adhesion molecules on cDCs with the respective cognate receptors on T cells is required. Together, they form an immunological synapse of concentric regions termed supramolecular activation clusters (SMAC) [31,32]. Given that we found strongly reduced MHC-II and CD40 expression in C5aR1^+^ cDCs, we postulated that the interaction between OVA-specific TCR tg T cells and C5aR1^+^ cDCs might be reduced as compared to C5aR1^−^ cDCs. To study cDC/T cell interactions in vitro, we labeled FACS-purified cDCs from allergen-immunized WT mice with PKH-26 and T cells with CFSE and monitored their interactions for 300 min by confocal microscopy (Figure 4A–C).

We found that the number of interactions between T cells and C5aR1^+^ cDCs during the first 2 h was much lower than that of C5aR1^−^ cDCs, strongly indicating that the low MHC-II expression in C5aR1^+^ cDCs prevented optimal antigen presentation via MHC-II and sustained interaction via CD40. Additionally, at the end of the observation period (260–300 min), a marked reduction of CD4^+^ T cell interaction with C5aR1^+^ cDCs occurred, whereas the high level of interaction was unchanged between C5aR1^−^ cDCs and CD4^+^ T cells. Together, these data suggest that TCR tg CD4^+^ T cells only transiently engage with C5aR1^+^ cDCs and that this engagement is less stable than that with C5aR1^−^ cDCs.

### 3.5. C5aR1^+^cDCs Express Significantly Lower Levels of CCR7 in Comparison to C5aR1^−^ cDCs

**Figure 5:**
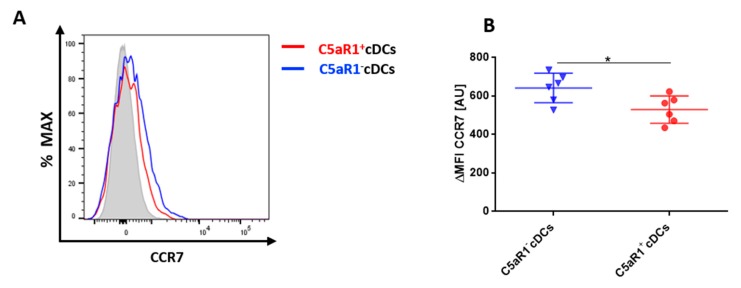
CCR7 expression in pulmonary C5aR1^+^ and C5aR1^−^ cDCs (**A**) Histogram showing the expression of CCR7 in C5aR1^+^ and C5aR1^−^ cDCs FACS-sorted 24 h after i.t. HDM/OVA immunization (100 μg/40 μg). We determined 10,000 events for each of the two subpopulations. The gray histogram represents the FMO control. (**B**) Quantitative assessment of CCR7 expression in C5aR1^−^ and C5aR1^+^ cDCs. Shown is the ΔMFI for CCR7 within the two subsets. The ΔMFI is defined as the mean fluorescence intensity of the signal normalized to the FMO control. Data shown are the mean ± SEM, *n* = 6 per group, Differences between groups were assessed by unpaired t-test, * *p* < 0.05.

To activate T cells, pulmonary cDCs need to migrate to the draining lymph nodes (dLNs). To compare the potential migratory capacity of the two CD11b^+^ cDC subsets, we determined the expression of CCR7, which is upregulated on activated DCs and is critical for their migration to the dLNs [6]. CCR7 recognizes CCL19 and CCL21, two chemokines that are produced in the lymph nodes and increase the expression of MHC-II and co-stimulatory molecules on DCs [6]. The expression of CCR7 was tested 24 h after i.t. HDM/OVA exposure (Figure 5). C5aR1^−^ cDCs expressed significantly higher levels of CCR7 in comparison to the C5aR1^+^ cDCs (Figure 5A,B). Given that CCR7 is critical for migration into the dLNs, these findings may suggest that C5aR1^−^ cDCs migrate more efficiently to the dLNs, where they may interact more efficiently with T cells due to their higher expression levels of MHC-II and CD40.

### 3.6. In Vitro Targeting of C5aR1 Increases the Potency of C5aR1^+^ cDCs to Induce CD4^+^ T Cell Proliferation through Regulation of CD40 Expression

**Figure 6:**
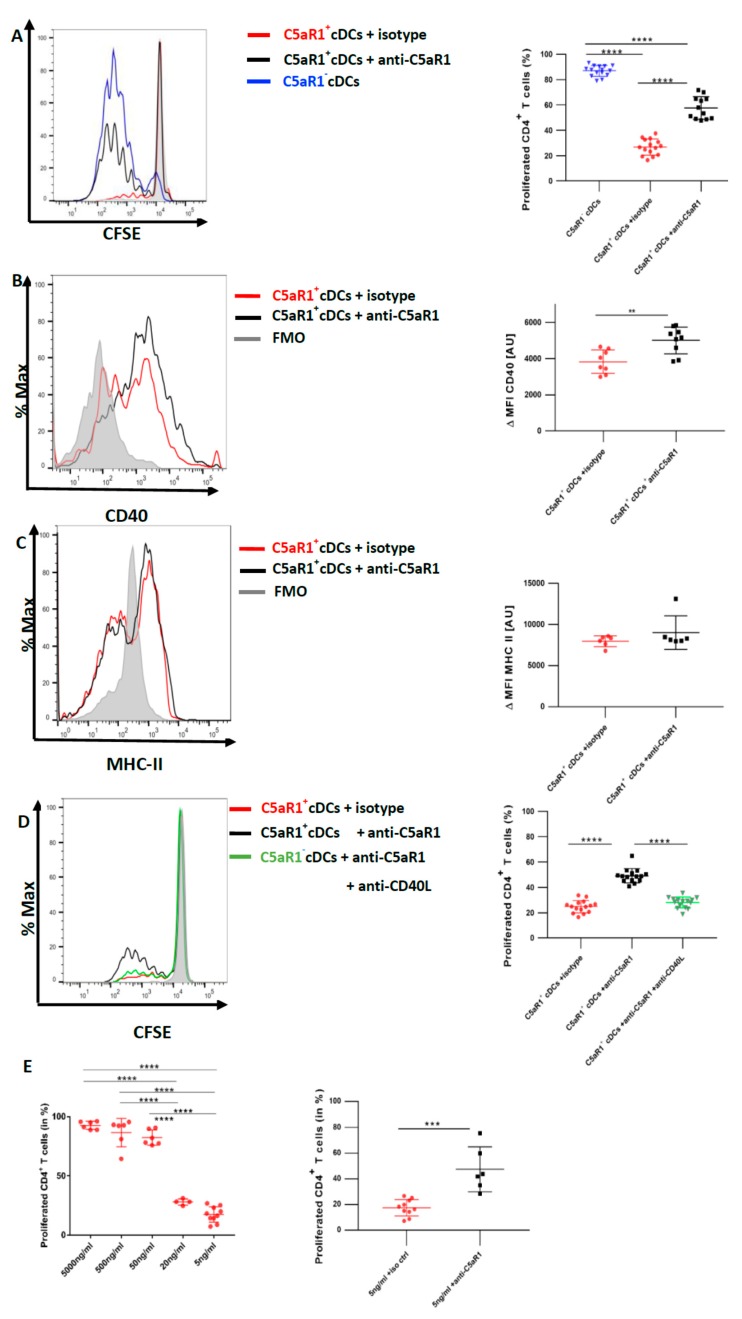
In vitro targeting of C5aR1 strongly enhances the potency of C5aR1^+^ cDCs to promote CD4^+^ T cell proliferation through upregulation of CD40. WT mice were treated once with a combination of HDM/OVA (100 μg/40 μg) i.t. C5aR1^+^ and C5aR1^−^ CD11b^+^ cDCs from WT mice were FACS-purified from lung tissue 24 h after the treatment. C5aR1^+^ cDCs were either treated with 5 μg/mL of the C5aR1-specific neutralizing mAb 20/70 or an appropriate isotype control. (**A**) cDCs were co-cultured with CFSE-labeled OVA-specific TCR tg CD4^+^ T cells in the presence of OVA (10 μΜ) and GM-CSF (20 ng/mL) for four days. Left panel; histogram detailing the T proliferation in the different treatment groups; the grey histogram shows the signal obtained from T cell directly after CFSE labeling; right panel: quantification of T cell proliferation in the different treatment groups. Values shown are the mean ± SEM; *n* = 12. Data were analyzed by ANOVA, followed by Tukey’s posthoc test; * indicates significant differences between the different treatment groups, **** *p* < 0.0001. (**B**,**C**) Evaluation of CD40 (**B**) and MHC-II (**C**) expression 18 h after in vitro targeting C5aR1. Left panels: histograms showing the expression of CD40 (**B**) or MHC-II (**C**) in the presence or absence of C5aR1 targeting, grey histogram = FMO control; right panels: quantitative assessment of CD40 or MHC-II expression in the presence or absence of C5aR1 targeting. Shown is the expression of CD40 or MHC-II (as ΔMFI) in the two treatments groups as mean ± SEM; the ΔMFI is defined as the mean fluorescence intensity of the signal normalized to the FMO control, *n* = 6–9 per group. Data were analyzed with unpaired t-test; * indicates significance between the two treatment groups, ** *p* < 0.001. (**D**) Assessment of CD40L targeting on T cell proliferation in C5aR1^+^ cDCs, in which C5aR1 signaling was blocked. Left panel: histogram showing the impact of C5aR1 blockade vs. C5aR1 and CD40/CD40L blockade on T cell proliferation; the grey histogram shows the signal obtained from T cell directly after CFSE labeling. The graph on the right side shows the quantitative evaluation of the different treatment groups. Values shown are the mean ± SEM; *n* = 15 per group. Data were compared by ANOVA followed by Tukey posthoc test. * indicates significant differences between the treatment groups; **** *p* < 0.0001. (**E**) Impact of C5aR1 targeting on T cell proliferation when the antigen concentration is limited. WT mice were treated once with HDM (100 μg) i.t. C5aR1^+^ cDCs from WT mice were FACS-purified from lung tissue 24 h after the treatment. C5aR1^+^ cDCs were co-cultured with CFSE-labeled OVA-specific TCR tg CD4^+^ T cells in the presence of different concentrations of OVA^323–339^ peptide (5 μg/mL, 500 ng/mL, 50 ng/mL, 20 ng/mL and 5 ng/mL) and GM-CSF (20 ng/mL). Left panel: frequency of proliferated T cells in response to different concentrations of OVA^323−339^ peptide as mean ± SEM, *n* = 3–9. Data were analyzed by ANOVA, followed by Tukey post-hoc test; * indicates significant differences between the different treatment groups, **** *p* < 0.0001; right panel: impact of C5aR1 targeting on C5aR1^+^ cDC-driven T cell proliferation in the presence of low OVA^323–339^ peptide concentration, *n* = 6–10. Data were analyzed by unpaired t test; * indicates significant differences between the two treatment groups; *** *p* < 0.001.

To directly determine the impact of C5aR1 signaling in C5aR1^+^ cDCs on T cell proliferation, we blocked C5aR1 using a neutralizing Ab against the receptor in vitro [11]. More precisely, 24 h after HDM/OVA immunization, C5aR1^+^ cDCs were FACS-purified and in vitro pulsed with OVA in the presence or absence of a C5aR1-neutralizing mAb. Eighteen hours later, both groups of C5aR1^+^ cDCs were co-cultured with CFSE-labeled-CD4^+^ T cells from OVA-specific TCR Tg DO11.10 RAG2^−/−^ mice. Four days later, we evaluated the T cell proliferation. In vitro targeting of C5aR1 significantly restored the potency of C5aR1^+^ cDCs to induce CD4^+^ T cell proliferation. In more detail, upon in vitro C5aR1 blockade, the frequency of activated CD4^+^ T cells increased to 60 ± 10% in contrast to the treatment with the isotype control, which resulted in activation of 30 ± 10% of the CD4^+^ T cells. These findings demonstrate that signaling through C5aR1 is an important mechanism to control CD4^+^ T cell proliferation by pulmonary CD11b^+^ cDCs (Figure 6A).

Given that MHC-II and CD40 were decreased in C5aR1^+^ cDCs after HDM/OVA immunization in comparison to C5aR1^−^ cDCs (Figure 3), we next evaluated the effect of in vitro blockade of C5aR1 on the expression levels of MHC-II and the co-stimulatory molecule CD40 on C5aR1^+^ cDCs. We assessed MHC-II and CD40 expression 18 h after in vitro blocking of C5aR1 and before adding the T cells to the system (Figure 6B,C). We noticed a significant up-regulation of CD40, while the expression of MHC-II was unchanged. These findings demonstrate that activation of C5a/C5aR1 axis suppresses the expression of CD40 suggesting that CD40 may control CD4^+^ T cell proliferation.

To test this hypothesis, we blocked the CD40-CD40L interactions using a neutralizing Ab against CD40L in C5aR1^+^ cDCs, in which C5aR1 was targeted by a neutralizing mAb. As shown in Figure 6A, blockade of C5aR1 markedly increased the potency of C5aR1^+^ cDCs to drive T cell proliferation. When C5aR1 and CD40L were simultaneously targeted in vitro, we noticed a decreased potency of the C5aR1^+^ cDCs to induce CD4^+^ T cell proliferation (Figure 6D). In fact, the T cell proliferation was as low as the one observed in untreated C5aR1^+^ cDCs (Figure 2A). These data demonstrate that the CD40-CD40L interaction in C5aR1^+^ cDCs is critical for strong T cell proliferation.

### 3.7. CD40-CD40L Interaction Controls the Proliferation of CD4^+^ T Cells When the Availability of the Antigen Peptide Is Limited on C5aR1^+^ cDCs 

CD40 is an important regulator of B cell functions [33]. Targeting the CD40/CD40 ligand (CD154) interaction by CD40 siRNA given to bone-marrow-derived DCs attenuated allergic rhinitis and induced the conversion of CD4^+^ T cells into regulatory T cells [34]. However, less is known about the role of CD40 as a regulator of T cell proliferation. To better understand the conditions under which CD40 activation on C5aR1^+^ cDCs regulates T cell proliferation, we pulsed FACS-purified cells in vitro with OVA^323−339^peptide, which specifically binds to the TCR of T cells from DO11.10 RAG2^−^/^−^ mice. We used five different concentrations of the peptide ranging from 5 μg/mL to 5 ng/mL. Interestingly, when the cells were pulsed with high OVA^323−339^ peptide concentrations (5 μg/mL and 500 ng/mL), C5aR1^+^ cDCs induced a robust T cell proliferation similar to that observed with C5aR1^−^ cDCs (Figure 6E left panel, and Figure 2B). However, when we reduced the concentration of the OVA^323−339^ peptide (50 ng/mL, 20 ng/mL and 5 ng/mL), the frequency of proliferated CD4^+^ T cells gradually declined to 20% (Figure 6E left panel), which is similar to what we had observed when C5aR1^+^ cDCs were pulsed with the whole OVA allergen (Figure 2A). Next, we tested if anti-C5aR1-treatment of C5aR1^+^ cDCs pulsed with 5 ng/mL of OVA^323−339^ peptide would enhance T cell proliferation. Clearly, C5aR1^+^ cDC targeting enhanced T cell proliferation (Figure 6E right panel). These data suggested that CD40 tips the balance of T cell proliferation only when a limited number of MHC-II molecules are loaded with antigen.

### 3.8. CD40-CD40L Interaction Controls CD4^+^ T Cell Proliferation in C5aR1^−^ cDCs When the Number of Antigen Peptide-Loaded MHC-II Molecules Is Low

**Figure 7:**
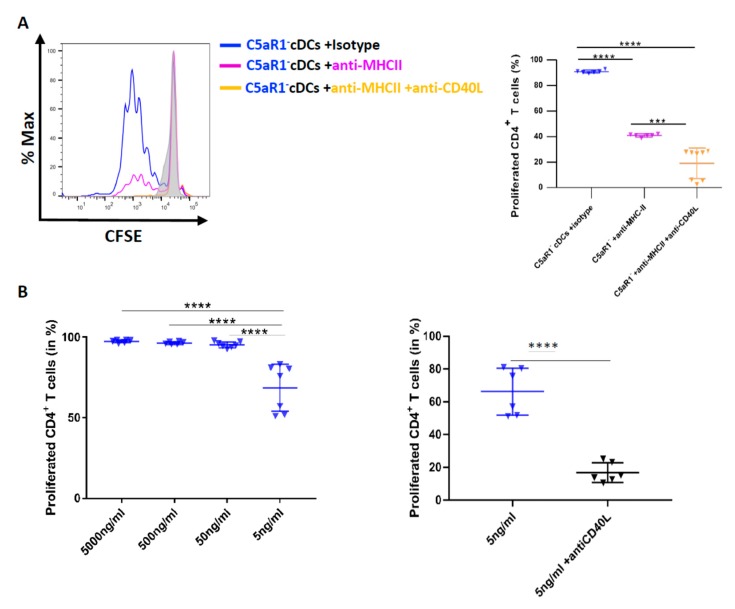
The effect of MHC-II targeting on C5aR1^−^ cDC-driven T cell proliferation in response to CD40L neutralization. (**A**) Histogram (left) showing the impact of MHC-II (10 pg/mL) blockade on T cell proliferation in C5aR1^−^ cells in the presence or absence of CD40L blockade; the grey histogram shows the signal obtained from T cell directly after CFSE labeling. The graph on the right-hand side shows the quantitative evaluation of MHC-II ± CD40L blockade. Values shown are the mean ± SEM; *n* = 6–8 per group. Data were analyzed by ANOVA, followed by Tukey post-hoc test; * indicates significant differences between the treatment groups; *** *p* < 0.001 and **** *p* < 0.0001. (**B**) Impact of CD40L targeting on T cell proliferation when the antigen concentration is limited. WT mice were treated once with HDM (100 μg) i.t. C5aR1^−^ cDCs from WT mice were FACS-purified from lung tissue 24 h after the treatment. C5aR1^−^ cDCs were co-cultured with CFSE-labeled OVA-specific TCR tg CD4^+^ T cells in the presence of different concentrations of OVA^323–339^ peptide (5 μg/mL, 500 ng/mL, 50 ng/mL, 20 ng/mL and 5 ng/mL) and GM-CSF (20 ng/mL). Left panel: frequency of proliferated T cells in response to different concentrations of OVA^323–339^ peptide as mean ± SEM, *n* = 6–7. Data were analyzed by ANOVA, followed by Tukey post-hoc test; * indicates significant differences between the different treatment groups, **** *p* < 0.0001; right panel: impact of CD40L targeting on C5aR1^−^ cDC-driven T cell proliferation in the presence of low OVA^323–339^ peptide concentration, *n* = 6–7. Data were analyzed by unpaired t test; * indicates significant differences between the two treatment groups; **** *p* < 0.0001.

With the above data in mind, we postulated that we should also be able to reduce the strong potency of C5aR1^−^ cDCs to drive T cell proliferation by targeting MHC-II and further reduce it by additional targeting of CD40L. Thus, we used FACS-purified C5aR1^−^ cDCs from HDM/OVA immunized mice and first blocked MHC-II using different concentrations of a neutralizing MHC-II-specific mAb (data not shown). Eventually, we found that 10 pg/mL of the neutralizing MHC-II specific mAb reduced the potency of C5aR1^−^ cDCs to promote T cell proliferation from >90% to 40% (Figure 7A right panel). Next, we simultaneously treated the cells with neutralizing Abs against MHC-II and CD40L.

Importantly, CD40L targeting further decreased T cell proliferation to <20% (Figure 7B). These data suggest that anti-CD40L treatment was able to markedly reduce T cell proliferation in C5aR1^-^ cDCs, when the number of OVA-peptide-loaded MHC-II molecules available for TCR interaction were limited. To further test this hypothesis, we pulsed C5aR1^−^ cDCs with different concentrations of OVA^323−339^ peptide (from 5 μg/mL to 5 ng/mL). When the availability of the OVA^323−339^ peptide was limited (5 ng/mL), the proliferation of OVA-specific TCR Tg CD4^+^ T cells was reduced to 70% (Figure 6C left panel), which was similar to what we had observed when C5aR1^+^ cDCs were loaded with 50 ng/mL of OVA peptide (Figure 6E left panel). When we treated the C5aR1^−^ cDCs with 5 ng/mL OVA^323−339^ peptide in the presence of the CD40L specific mAb, the potency of C5aR1^−^ cDCs to drive T cell proliferation dropped to <20% (Figure 7B right panel).

Taken together, our findings strongly suggest that CD40/CD40L interaction is a critical factor for T cell proliferation when MHC-II expression or antigen-loading is low and that C5a/C5aR1 axis activation in C5aR1^+^ cDCs controls T cell proliferation through its impact on CD40 expression (Figure 6B).

### 3.9. Allergen Stimulation of C5aR1^+^ and C5aR1^−^ cDCs Induces the Production of C5 and Cleavage into C5a

**Figure 8:**
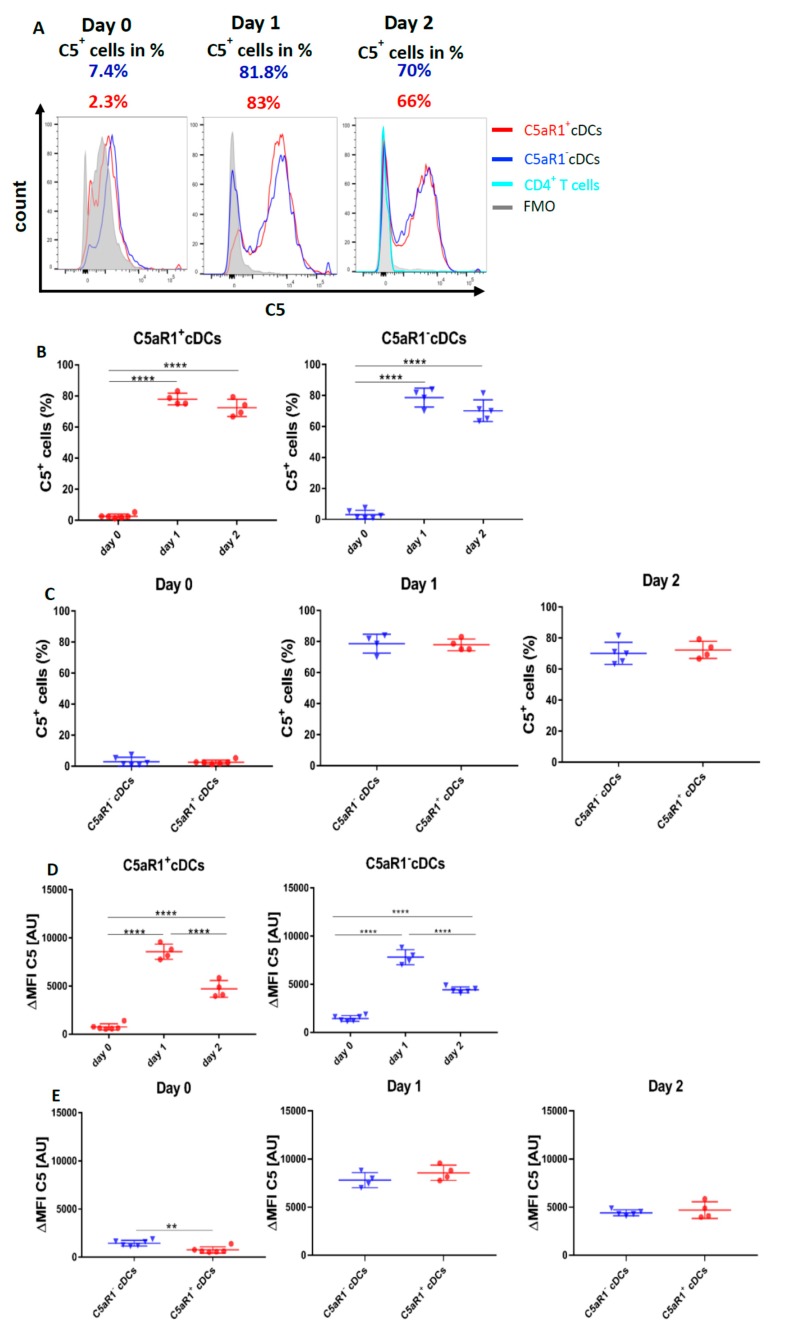

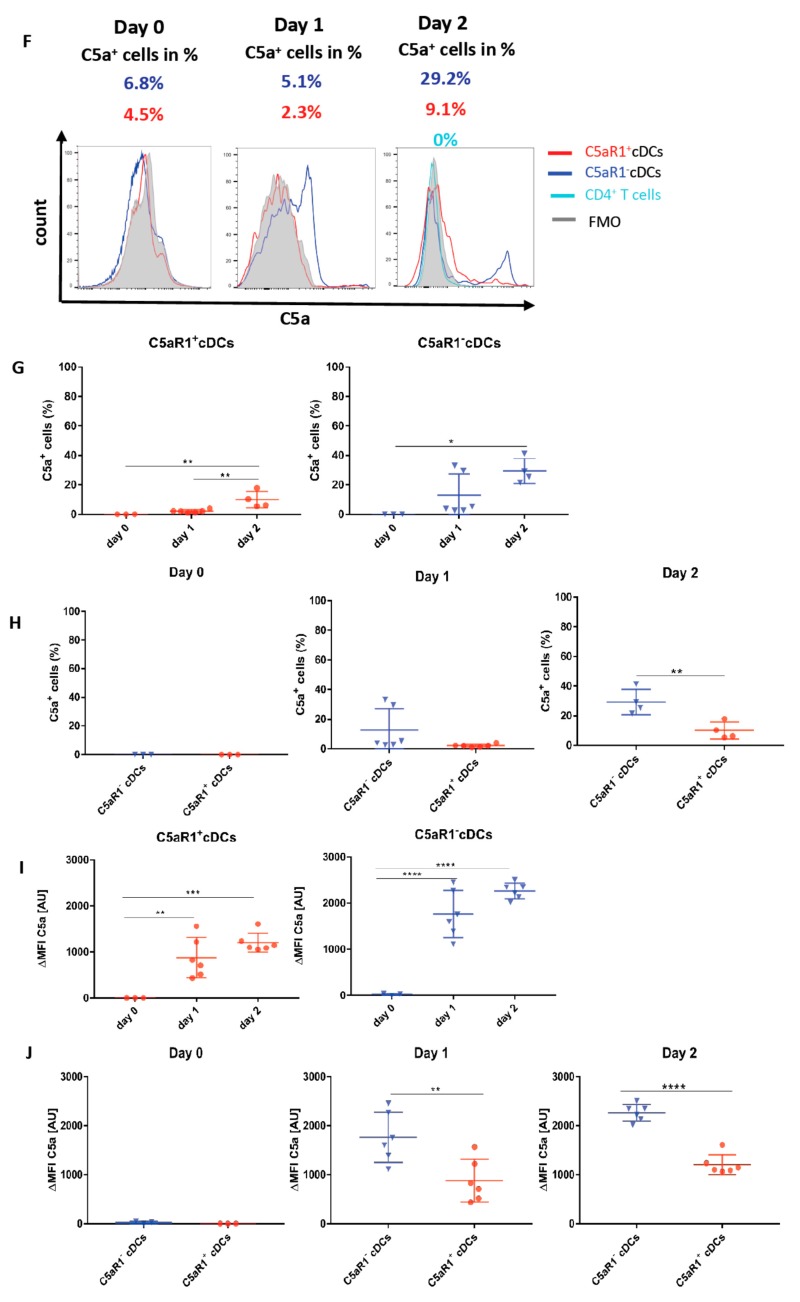

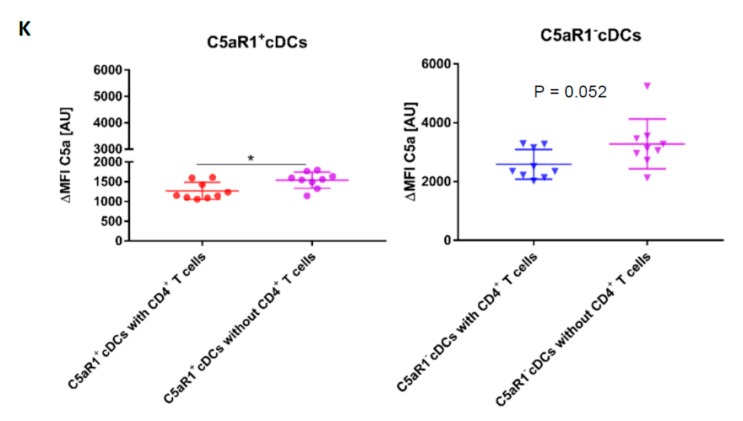
Impact of OVA-pulsing and T cell co-culture on C5 production and C5a generation from C5aR1^+^ and C5aR1^−^ cDCs. (**A**,**F**) Histograms showing C5 production (**A**) or C5a generation (**F**) in C5aR1^+^ or C5aR1^−^ cDC subsets directly after FACS purification on day 0, after OVA-pulsing on day 1 or after addition of OVA-tg CD4^+^ T cells on day 2; grey histogram = FMO control. (**B**,**G**) Frequency of C5-producing (**B**) or C5a-generating (**G**) C5aR1^+^ (left panel) or C5aR1^−^ cDCs (right panel). (**C**,**H**) Comparison of the frequencies of C5-producing (**C**) or C5a-generating (**H**) C5aR1^+^ and C5aR1^−^ cDCs on days 0, 1 and 2. (**D**,**I**) Quantitative evaluation of C5 production (**D**) or C5a generation (**I**) in C5aR1^+^ (left panel) or C5aR1^−^ (right panel) cDCs on days 0, 1 and 2. (**E**,**J**) Comparison of C5 production (**E**) or C5a-generation (**J**) in C5aR1^−^ or C5aR1^+^ cDCs on days 0, 1 and 2. The data shown in D, E, I and J show the ΔMFI of C5 or C5a expression by the two cDC subsets; the ΔMFI is defined as the mean fluorescence intensity of the signal normalized to the FMO control. Data shown in B–E and G–J are the mean ± SEM, *n* = 4–6 per group; data in B, D, G and I were analyzed by ANOVA followed by Tukey’s post-hoc test; * indicates significant differences between C5aR1^+^ and C5aR1^−^ cDCs on day 0 vs. days 1 and 2; ** *p* < 0.01, *** *p* < 0.001, **** *p* < 0.0001; data in C, E, H and J were compared by unpaired t-test; * indicates significant differences between C5aR1^+^ and C5aR1^−^ cDCs on days 0, 1 or 2; ** *p* < 0.01, **** *p* < 0.0001. (**K**) Impact of T cells on OVA-driven C5a production C5aR1^+^ (left panel) or C5aR1^−^ (right panel) cDCs from allergen-exposed mice. Shown is the intracellular expression of C5a in OVA-pulsed cDCs that were co-cultured with or without OVA-specific TCR tg CD4^+^ T cells as ΔMFI of C5a expression by the two cDC subsets. Data shown are the mean ± SEM. *n* = 9. They were analyzed by unpaired t-test. * *p* < 0.05.

Our findings that C5aR1^+^ and C5aR1^−^ cDCs exert a distinct potency to drive T cell proliferation and differentiation in vitro in co-cultures with CD4^+^ T cells suggests that C5aR1 is activated during the co-culture to promote the observed effects. However, at this point, neither the source (cDC and/or T cell) nor the time of C5 production and cleavage into C5a was clear. To determine the source of C5, we determined intracellular C5 production in C5aR1^−^ and C5aR1^+^ cDCs on day 0, i.e., when cells were sorted after HDM/OVA exposure; on day 1, when cDCs were in vitro pulsed with OVA, but before the T cells were added to the system; and on day 2, i.e., when DCs and T cells had interacted for 24 h hours (Figure 8).

On day 0, around 2.3% of C5aR1^+^ and 7.4% of C5aR1^−^ cDCs (Figure 8A–C) produced C5 directly after sorting. After OVA-pulsing on day 1, the frequency of C5-producing C5aR1^+^ and C5aR1^−^ cDCs markedly increased. In contrast to day 0, 83% of C5aR1^+^ and 82% of C5aR1^−^ cDCs produced C5. After addition of T cells on day 2, the frequency of C5 producing C5aR1^+^ and C5aR1^−^ cDCs slightly decreased to 66% of C5aR1^+^ and 71% of C5aR1^−^ cDCs. Importantly, the T cells did not produce any C5. To determine the individual C5 expression in C5aR1^+^ and C5aR1^−^ cDCs on days 0, 1 and 2, we compared the ΔMFI. As shown in Figure 8G, C5 production was significantly higher in C5aR1^−^ cDCs than in C5aR1^+^ cDCs on day 0. In contrast, C5 production increased to the same level in C5aR1^+^ and C5aR1^−^ cDCs on day 1 and the expression levels in both cDC subsets were also similar on day 2. These findings demonstrate that OVA-pulsing is an important inducer of C5 production in C5aR1^+^ and C5aR1^−^ cDCs and that C5aR1^+^ and C5aR1^−^ cDCs strongly reduce their C5 production after T cell interaction.

Next, we determined the impact of OVA-pulsing and DC/T cell interaction on intracellular C5a generation from cDC-produced C5. On day 0, none of the two CD11b^+^ cDC subsets produced detectable C5a (Figure 8F–H). Only after in vitro pulsing with OVA, the cDCs started to generate C5a. In C5aR1^+^ cDCs, 2.3% of cells produced C5a, whereas in C5aR1^−^ cDCs, 5.1% were C5a producers. Strikingly, the C5a production significantly increased after the addition of T cells on day 2 (Figure 8F–H). Around 9.1% of C5aR1^+^ and 29.2% of C5aR1^−^ cDCs produced C5a. The higher frequency of C5a^+^ cells within the C5aR1^−^ cDC subpopulation may result from the lack of C5a/C5aR1 binding in the absence of C5aR1 expression. In accordance with our findings that T cells produce no C5, we also found no production of C5a (Figure 8F).

To directly assess the impact of T cells on C5a production from CD11b^+^ cDCs, we compared the C5a generation on day 2 in the presence or absence of CD4^+^ T cells. As shown in Figure 8K, we found a stronger allergen-induced C5a generation from C5 in C5aR1^+^ cells in the absence of CD4^+^ T cells and a similar trend for C5aR1^−^ cDCs. Together, the data suggest that similar to the suppression of C5 production, CD4^+^ T cells also suppress the generation of C5a from C5. However, this effect seems not to be as pronounced as for C5.

## 4. Discussion

Previous studies have shown that the C5/C5a/C5aR1 axis activation during allergen sensitization controls the development of AHR, mucus production, airway inflammation and the development of maladaptive Th2/Th17 development [11,12,16,17]. These findings suggest that C5aR1 controls the development of maladaptive Th2/Th17 responses at several levels. First, it tips the balance between immunogenic pulmonary CD11b^+^ cDCs and tolerogenic pDCs [11]. Second, it regulates co-stimulatory molecule PD-L1 and PD-L2 expression on pDCs [13]. By both mechanisms, it regulates Th2 cytokine production from CD4^+^ T cells and controls expansion of myeloid suppressor cells from bone marrow and their subsequent activation [14]. However, it is still unknown if direct C5aR1 activation on pulmonary CD11b^+^ cDCs regulates their ability to activate naive CD4^+^ T cells during allergen sensitization.

Studies of the Lambrecht group identified CD11b^+^ CD64^−^ cDCs as the main migratory subset, which drives Th2/Th17 cell mediated immunity in the lymph nodes [4]. They identified such cells as the dominant cell type that took up antigen and migrated to the draining lymph nodes to activate naïve CD4^+^ T cells. Using a GFP-C5aR1 knock-in mouse, we found that some but not all CD11b^+^ cDCs express C5aR1 in the lung of C57BL/6 mice [22]. We hypothesized that C5aR1 activation in CD11b^+^ cDCs controls the potency of these cells to activate T cells. To test this hypothesis, we first determined the expression of C5aR1 in pulmonary CD11b^+^ cDCs, both under steady-state conditions and after one-time HDM/OVA exposure. As expected, we observed that the CD11b^+^ CD64^−^ cDC population expressed C5aR1 to a different degree. In naïve BALB/c mice, the majority of the CD11b^+^ CD64^−^ cDCs (85%) were C5aR1^+^, whereas a minor fraction was C5aR1^−^ (15%). In response to HDM/OVA immunization, the frequency of the C5aR1^−^cDCs increased to 25%. The mechanisms driving the increased frequency of C5aR1^−^cDCs upon HDM/OVA exposure are unclear. First, naïve C5aR1^−^ cDCs may proliferate after HDM administration. However, this is unlikely as DCs have not been described to proliferate massively in tissues [35]. Alternatively, an increased recruitment to the lung tissue through CCR2 activation may occur [4]. Lastly, naïve C5aR1^+^cDCs may downregulate the expression of C5aR1 upon allergen exposure. We are currently in the process of generating C5aR1 fate mapping mice to address this possibility in future studies.

In addition to C5aR1 expression, we also determined the expression of CD24, CD301 and CD209, all under steady state conditions and after HDM/OVA exposure. We observed a heterogeneous expression pattern of these molecules within the CD11b^+^ cDC population demonstrating phenotypic heterogeneity of CD11b^+^ cDCs. Of note, CD209 expression negatively correlated with C5aR1 under steady conditions and also after HDM/OVA exposure.

Furthermore, we aimed to understand if the CD11b^+^ CD64^−^ C5aR1^+^ and CD11b^+^ CD64^−^ C5aR1^−^ cDCs were functionally different. First, we evaluated the potential of the two subsets to drive CD4^+^ T cell proliferation. Our data showed that the C5aR1^+^cDCs were poor inducers of CD4^+^ T cell proliferation as compared with C5aR1^−^ cDCs, which were very potent. This finding is of major interest as CD11b^+^ cDCs are the most potent pulmonary cDC population to drive T cell activation in response to allergen [4]. Given that the majority of CD11b^+^ cDCs express C5aR1, these data suggested that it is mainly the small C5aR1^−^ cDC population that efficiently promotes activation of naïve CD4^+^ T cell in response to allergen encounter, whereas the majority of the CD11b^+^ CD64^−^ cDCs that express C5aR1 play only a minor role.

The signals that lead to T-cell proliferation are generated at the level of the immunological synapse, a specialized area of contact between T cells and DCs. At the synapse, the TCRs are sequentially triggered by peptide-MHC complexes, a process that allows the signal to be sustained for as long as the synapse is in place. T cells continuously search for antigen and can rapidly move from one DC to another offering a higher level of stimulation. While the duration of TCR stimulation depends on the synapse, the intensity of the signal that T cells receive is dependent on both, the level of peptide-MHC complexes and the level of costimulatory molecules that amplify the signaling process [36,37,38]. Given that TCR/MHC-II and costimulatory interaction between DCs and T cells defines the formation of the immunological synapse, we determined MHC-II and costimulatory expression in C5aR1^+^ and C5aR1^−^ cDCs. Clearly, C5aR1^+^ cDCs expressed significantly lower levels of MHC-II and CD40 in DCs from naïve mice and after allergen exposure than C5aR1^−^ cDCs, thus, remaining in a less mature state, which might explain the lower potency of the C5aR1^+^ cDCs to drive efficient CD4^+^ T cell proliferation. It is well appreciated that the maturation status of DCs is critical for T cell activation. Immature DCs make only short low affinity contacts with T cells and do not induce TCR clustering, resulting in inefficient TCR signaling and abortive proliferation [39]. Our findings show that C5aR1^+^ cDCs were in a partially mature status. When we tracked the interactions between C5aR1^+^ or C5aR1^−^ cDCs with CD4^+^ T cells in vitro, we noticed that the C5aR1^−^ cDCs interacted with the T cells more frequently than the C5aR1^+^ cDCs. In particular during the first 50 min of DC/T cell co-culture, we found a markedly lower interaction of T cells with C5aR1^+^ cDCs, suggesting that C5aR1^+^ cDCs make only short contacts with T cell as compared with C5aR1^−^ cDCs. Together, our findings suggest C5aR1 activation on cDCs keeps the cells in a more immature state which prevents the efficient activation of naïve T cells. Importantly, several studies have shown that antigens targeted to immature DCs in lymph nodes induce peripheral tolerance [40] through Ag-specific T cell deletion [41,42]. Of note, we observed that the C5aR1- cDCs expressed significantly higher levels of CCR7 in comparison to the C5aR1^+^ cDCs, which suggests that C5aR1^−^ cDCs migrate more efficiently to dLNs where they can interact more efficiently with T cells due to their higher expression levels of MHC-II and CD40.

Secondly, we determined whether C5aR1^+^ and C5aR1^−^ cDCs differ in their potential to differentiate CD4^+^ T cell into Th2 or Th17 effector cells, both of which mediate the maladaptive immune response in HDM-mediated allergic asthma. We found that both cDC subsets drove a mixed Th2/Th17 response. C5aR1^−^ cDCs induced a slightly higher frequency of IL13^+^ Th2 effector cells than C5aR1^+^ (80% vs. 70%). In contrast, C5aR1^−^ cDCs were less potent in inducing IL-17A-producing Th17 cells than their C5aR1^+^ counterparts (5% vs. 10%). Taking into account that the frequency of proliferating T cells was much higher with C5aR1^−^ than with C5aR1^+^ cDCs, the overall frequency of proliferating Th2 (72% vs. 14%) and Th17 (4.5% vs. 2%) cells is much higher after exposure of T cells to C5aR1^−^ than to C5aR1^+^ cDCs. A different impact of BM-DCs [14,16] or spleen-derived DCs [43] from C5aR1-deficient- or WT mice on IL-12 family cytokine production has been shown before. IL-17A production was significantly reduced in *C5ar1^−/−^* BM-DC/T cell co-cultures as a result of deceased IL-23, IL-6 and IL-1β production [14], whereas Th2 cytokine production from CD4^+^ T cells was similar after in vitro OVA stimulation. In contrast, spleen-derived WT DCs switched from a Th1 to a Th17-inducing phenotype in the absence of C5aR1 signaling [43] demonstrating that the tissue origin of DC subsets is critical for C5aR1-induced differentiation pattern of naïve T cells.

To directly assess the role of C5aR1 on T cell proliferation, we blocked C5aR1 signaling in C5aR1^+^ cDCs in vitro using a C5aR1-neutralizing Ab. Strikingly, such treatment markedly increased the potency of C5aR1^+^ cDCs to drive CD4 T cell proliferation in comparison to the isotype treated C5aR1^+^ cDCs. Based on our findings that C5aR1^+^ cDCs express low levels of MHC-II and CD40, which could result in a compromised immunological synapse formation, we suspected that C5aR1 targeting impacts on MHC-II or CD40 expression. Interestingly, in vitro targeting of C5aR1 resulted in a significant upregulation of CD40 but had no impact on MHC-II expression suggesting that C5aR1 signaling in CD11b^+^ cDCs suppressed CD40 expression. Previous studies have shown that CD40/CD40L pairing occurs in the central region of the immunological synapse upon formation of the TCR/CD3 c-SMAC [37] and that CD40 clustering [44] is important in this process, at least in B cells. However, not many studies have focused on the role of CD40/CD40L interaction in the context of DC-driven activation of CD4^+^ T cells. When we simultaneously blocked C5aR1 and the CD40/CD40L interactions, we observed a significantly reduced T cell proliferation, strengthening the view that CD40/CD40L interaction is critical for T cell proliferation under conditions when MHC-II expression is low.

In an attempt to further support this view, we pulsed C5aR1^+^ cDCs with different doses of OVA^323−339^ peptide. While C5aR1^+^ cDCs were able to drive strong T cell proliferation at high peptide concentrations, it dropped to 20% when the OVA^323−339^ peptide concentration was low. In vitro blockade of C5aR1 under these conditions increased T cell proliferation to the same extent of what we had noted in the presence of whole OVA antigen. Furthermore, when we simultaneously targeted CD40L, T cell proliferation was markedly attenuated. Thus, under condition of limited antigen-peptide availability, CD40/CD40L interaction becomes critical for T cell proliferation. In support of this notion, we found a decreased potency of C5aR1^−^ cDCs, which express high MCH-II, to drive T cell proliferation when we targeted MHC-II or limited the amount of antigen peptide. Strikingly, we could even abolish T cell proliferation when we additionally target CD40/CD40L under such conditions.

Finally, we characterized the source of C5 and C5a as well as the mechanisms driving the production of C5 and consecutive cleavage into C5a to activate C5aR1 in C5aR1^+^ cDCs. We found that in vivo immunization induced C5 production in only a very minor fraction of C5aR1^+^ cDCs. This considerably changed on day 1, i.e., when the cells were in vitro pulsed with OVA allergen for 18 h, as almost 80% of both cDC subpopulation started to express C5. Interestingly, co-culture of cDCs with T cells resulted in a slight reduction in the percentage of C5^+^ cDCs suggesting some negative feedback of T cells to the cDCs. In addition to C5, C5aR1^+^ and C5aR1^−^ cDCs were able to cleave C5 into C5a intracellularly, as we found C5a in both cDCs subsets. The higher frequency of C5a^+^ cells in the C5aR1^−^ cDC subset might be explained by the lack of C5aR1 binding in such cells. Similar to C5 production, T cells had a negative effect on C5a generation, although it was not as pronounced. In future studies it remains to be determined which mechanism underly the cleavage of C5. Both canonical cleavage by formation of C5 convertases [45] as well as non-canonical cleavage by a cDC-derived protease need to be considered. Intracellular C5 generation and cleavage into C5a has been shown in human T cells [46] and mouse macrophages [10,47]. However, the nature of such proteases has not been uncovered.

## 5. Conclusions

**Figure 9:**
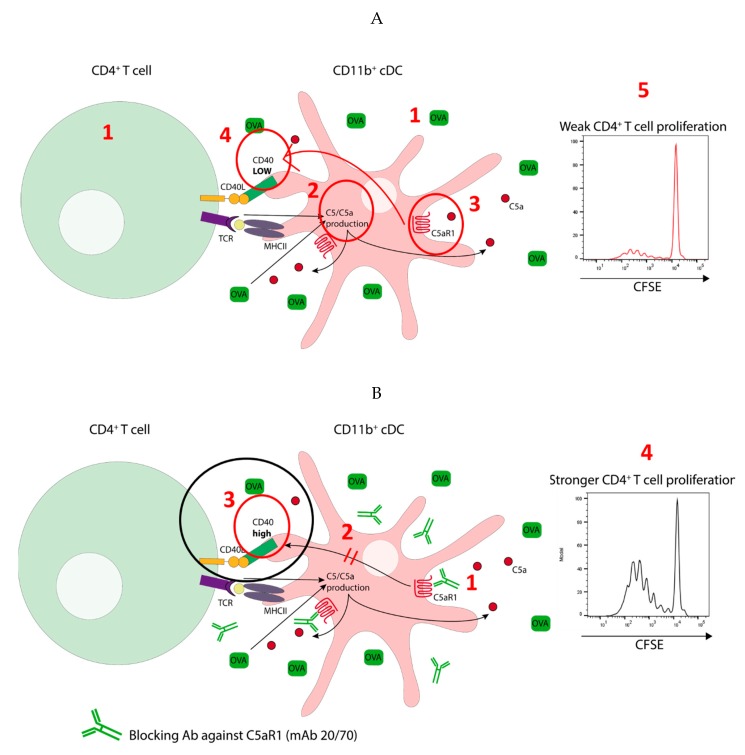
Model detailing the impact of C5a/C5aR1 axis activation in C5aR1^+^ cDCs on the activation of antigen specific CD4^+^ T cells. (**A**) Low potency of C5aR1^+^ cDCs to drive proliferation of antigen specific CD4^+^ T cells. C5a-mediated activation of C5aR1 suppresses the expression of CD40 in MHC-II^lo^ C5aR1^+^ cDCs. Under such conditions CD40 is critical for cDC-driven proliferation of antigen-specific T cells. (**B**) C5aR1-targeting of C5aR1^+^ cDCs increases their potency to drive CD4^+^ T cell proliferation. The lack of C5aR1 signaling results in increased CD40 expression in MHC-II^lo^ C5aR1^+^ cDCs leading to improved synapse formation and stronger T cell activation.

In summary, we propose two distinct pulmonary CD11b^+^ cDCs subsets in mice based on the expression of C5aR1, i.e., C5aR1^+^ and C5aR1^−^ cDCs. We found in a mouse model of one-time HDM/OVA allergen exposure that activation of C5aR1 on pulmonary CD11b^+^ cDCs has a strong impact on their function. As shown in Figure 9A, OVA antigen pulsing of CD11b^+^ cDCs (1) resulted in C5 production and consecutive C5a generation (2). Engagement of C5aR1 by C5a (3) kept the expression of CD40 low (4) and together with the low MHC-II expression in the C5aR1^+^ cDC subset, this resulted in impaired C5aR1^+^ cDC/CD4^+^ T cell interaction, eventually leading to minor proliferation of CD4^+^ T cells (5). As shown in Figure 9B, absence of C5aR1 activation in response to allergen exposure in C5aR1^−^ cDCs or in response to in vitro blockade of C5aR1 (1), released the break on CD40 expression (2), resulting in efficient interaction of CD11b^+^ cDCs with CD4^+^ T cells (3) as a prerequisite to drive strong CD4^+^ T cell proliferation (4).

Given that antigens targeted to immature DCs in lymph nodes induce peripheral tolerance [40] through Ag-specific T cell deletion [41,42] we suggest a model in which C5aR1 serves as a checkpoint in pulmonary CD11b^+^ cDC activation, which keeps them in a status of immaturity to protect the body from undesired activation of its most potent sentinels.
